# Association of amino acids and parameters of bone metabolism with endothelial dysfunction and vasculopathic changes in limited systemic sclerosis

**DOI:** 10.3389/fmed.2023.1193121

**Published:** 2023-06-23

**Authors:** Philipp Jud, Andreas Meinitzer, Heimo Strohmaier, Behrouz Arefnia, Gernot Wimmer, Barbara Obermayer-Pietsch, Vasile Foris, Gabor Kovacs, Balazs Odler, Florentine Moazedi-Fürst, Marianne Brodmann, Franz Hafner

**Affiliations:** ^1^Division of Angiology, Department of Internal Medicine, Medical University of Graz, Graz, Austria; ^2^Clinical Institute of Medical and Chemical Laboratory Diagnostics, Medical University of Graz, Graz, Austria; ^3^Center of Medical Research (ZMF), Medical University of Graz, Graz, Austria; ^4^Division of Restorative Dentistry, Endodontics, Periodontology and Prosthodontics, Department of Dental Medicine and Oral Health, Medical University of Graz, Graz, Austria; ^5^Division of Endocrinology and Diabetology, Department of Internal Medicine, Endocrinology Lab Platform, Medical University of Graz, Graz, Austria; ^6^Division of Pulmonology, Department of Internal Medicine, Medical University of Graz, Graz, Austria; ^7^Ludwig Boltzmann Institute for Lung Vascular Research, Graz, Austria; ^8^Division of Nephrology, Department of Internal Medicine, Medical University of Graz, Graz, Austria; ^9^Division of Rheumatology, Department of Internal Medicine, Medical University of Graz, Graz, Austria

**Keywords:** systemic sclerosis, endothelial dysfunction, amino acids, vitamin D, bone metabolism

## Abstract

**Objectives:**

Pathways contributing to endothelial dysfunction in patients with limited cutaneous systemic sclerosis (lcSSc) are largely unknown. The aim of this study was to investigate potential associations of amino acids and parameters of bone metabolism with endothelial dysfunction and vasculopathy-related changes in patients with lcSSc and early-stage vasculopathy.

**Methods:**

Amino acids, calciotropic parameters, including 25-hydroxyvitamin D and parathyroid hormone (PTH), and bone turnover parameters, including osteocalcin and N-terminal peptide of procollagen-3 (P3NP), were measured in 38 lcSSc patients and 38 controls. Endothelial dysfunction was assessed by biochemical parameters, pulse-wave analysis, flow-mediated and nitroglycerine-mediated dilation. Additionally, vasculopathy-related and SSc-specific clinical changes including capillaroscopic, skin, renal, pulmonary, gastrointestinal and periodontal parameters were recorded.

**Results:**

No significant differences in amino acids, calciotropic and bone turnover parameters were observed between lcSSc patients and controls. In patients with lcSSc, several significant correlations were found between selected amino acids, parameters of endothelial dysfunction, vasculopathy-related and SSc-specific clinical changes (all with *p* < 0.05). In addition, significant correlations were observed between PTH and 25-hydroxyvitamin D with homoarginine, and between osteocalcin, PTH and P3NP with modified Rodnan skin score and selected periodontal parameters (all with *p* < 0.05). Vitamin D deficiency defined as 25-hydroxyvitamin D < 20 ng/ml was associated with the presence of puffy finger (*p* = 0.046) and early pattern (*p* = 0.040).

**Conclusion:**

Selected amino acids may affect endothelial function and may be associated to vasculopathy-related and clinical changes in lcSSc patients, while the association with parameters of bone metabolism seems to be minor.

## Introduction

Systemic sclerosis (SSc) represents a rare autoimmune connective tissue disease with potentially serious complications due to its effects on skin and internal organs. There are three hallmarks in the pathogenesis of SSc, including autoimmune activation, tissue fibrosis and vasculopathy, while vasculopathy seems to occur predominantly in limited cutaneous SSc (lcSSc) (1–3). Due to interactions between different cells types, extracellular matrix and cytokines, structural and functional changes of micro-and macrovascular vessels occur. They lead to clinical manifestations of SSc, including Raynaud’s phenomenon, capillary changes or pulmonary arterial hypertension (PAH) (3–5). Besides these commonly vasculopathy-driven symptoms and complications, vasculopathy and endothelial dysfunction may also contribute to other manifestations of SSc, like skin, renal and gastrointestinal involvement or periodontal disease (6–9).

Multiple parameters of endothelial dysfunction are known so far, including pulse-wave velocity (PWV), flow-mediated dilation (FMD), nitroglycerine-mediated dilation (NMD), biochemical parameters of the arginine metabolism or endothelial microparticles (EMP), which have also been evaluated in SSc (10–12). However, potential contributors to endothelial dysfunction and vasculopathic changes in SSc are still largely unknown. While both arginine and homoarginine have been investigated as potential contributors to endothelial dysfunction in SSc, the knowledge on potential influences of other amino acids on parameters of endothelial dysfunction and vasculopathic changes in SSc are limited (12–14). Another extensively investigated metabolic parameter is vitamin D and vitamin D deficiency is commonly found in patients with SSc, which may also be associated with SSc-related complications (15). Especially, as vitamin D has immunomodulatory effects by inhibition of T helper-1 lymphocytes and proinflammatory cytokines on the one hand and promotion of anti-inflammatory cytokines on the other hand, these properties may contribute to SSc-related complications (16). Additionally, vitamin D also exhibits antifibrotic properties, with a reported downregulation of several profibrotic biomarkers, and cardioprotective effects by downregulation of prothrombotic factors and exerting anti-hypertrophic effects on cardiomyocytes (16–18). However, vitamin D revealed inconsistent findings in endothelial dysfunction, also in SSc (19–21). Other parameters of bone metabolism, such as parathyroid hormone (PTH) and osteocalcin, may also affect endothelial dysfunction potentially by oxidative stress (22, 23). However, data about the interaction of bone metabolism on endothelial dysfunction in SSc are still lacking.

This study aimed to evaluate associations between proteinogenic amino acids, calciotropic and bone turnover parameters with endothelial dysfunction and vasculopathy-related changes in patients with lcSSc.

## Materials and methods

### Study design and patient cohort

As this study is a sub-analysis of a prior case–control study of lcSSc patients in order to investigate endothelial dysfunction at early-stage vasculopathy, full details about the study design and parameter measurements have been previously described (12). Briefly, patients with lcSSc and a control group of age-, race-and sex-matched subjects with primary Raynaud’s phenomenon were included. Inclusion criterion for the group of patients with lcSSc was the presence of diagnosed lcSSc according to the EULAR/ACR criteria of 2013 (2). Exclusion criteria for both groups were age < 18 years, presence of diffuse cutaneous SSc or other connective tissues diseases, preexisting or existing PAH, digital ulcers, endoscopic approved reflux, diabetes mellitus or symptomatic atherosclerotic cardiovascular diseases, recent pregnancy or malignancies, acute infections at time of enrolment and current intake (<24 h) of prostanoids, calcium channel blockers, phosphodiesterase-5 inhibitors or endothelin-receptor inhibitors. Primary endpoint was the difference of amino acids and metabolic bone parameters between patients with lcSSc and controls. Secondary endpoints were associations between amino acids and metabolic bone parameters with parameters of endothelial dysfunction and vasculopathy-related as well as SSc-specific clinical changes.

### Evaluation of amino acids, calciotropic and bone turnover parameters

Fasting blood samples were obtained by standard phlebotomy during study inclusion. Following amino acids were assessed by high-performance liquid chromatography: alanine, asparagine, aspartic acid, glutamine, glutamic acid, glycine, histidine, isoleucine, leucine, lysine, methionine, phenylalanine, serine, threonine, tryptophan, tyrosine, and valine. Proline and cysteine could not be measured by this method, and taurine was evaluated instead. 25-hydroxyvitamin D [25-(OH)-D] and PTH were measured as calciotropic parameters, N-terminal peptide of procollagen-3 (P3NP) and osteocalcin were measured as bone turnover parameters by routine laboratory work-up. Vitamin D insufficiency was defined as serum 25-(OH)-D levels <30 ng/ml, vitamin D deficiency was defined as serum 25-(OH)-D levels <20 ng/ml.

### Evaluation of endothelial dysfunction

Arterial stiffness was evaluated by automated pulse-wave analysis via an oscillometric device (I.E.M. Mobil-O-Graph, I.E.M., Stolberg, Germany) while vascular reactivity was measured by FMD and NMD according to the guidelines by Corretti et al. (24). PWV of >10 m/s, FMD < 7% and NMD < 15.6% were defined as pathologic (25–27). Parameters of arginine metabolism, including asymmetric dimethylarginine (ADMA), symmetric dimethylarginine (SDMA), homoarginine, arginine, citrulline and ornithine, were measured by high-performance liquid chromatography. Measurement of EMP was performed according to the recommendations published by Cossarizza et al. (28) identifying EMP as CD31+/CD42b-events (29). Detailed information about the measurement methods of the respective parameters of endothelial dysfunction have been previously published by Jud et al. (12).

### Vasculopathy-related and SSc-specific clinical changes

Capillaroscopic changes were evaluated by nailfold videocapillaroscopy (NVC) of the second to fifth digit on both hands (Skinview, Optometron Ltd., Oskar-Messterstr., Ismaning, Germany). Morphological changes of the capillaries including SSc-specific capillary patterns, capillaroscopic skin ulcer risk index (CSURI) and microangiopathy evolution score (MES) were recorded (30, 31). Skin involvement, including telangiectasia, puffy finger, sclerodactyly, and modified Rodnan Skin Score (mRSS) were recorded by physical examination. Renal involvement was evaluated by estimated glomerular filtration rate (eGFR) with CKD-EPI equation and urinary total protein/creatinine ratio. Evaluation of subclinical PAH was performed by the DETECT algorithm while evaluation of gastrointestinal involvement was performed by UCLA SCTC GIT 2.0 questionnaire (32, 33). Disease severity was evaluated by the revised European Scleroderma Trials and Research Group (EUSTAR) index (34). Parameters of periodontal disease, including pocket depth, plaque index, bleeding on probing (BOP), bone loss and bone support ratio were evaluated by dental and oral examinations with a dentist’s mirror and a periodontal probe as well as by digital dental panoramic radiograph (Orthophos XG 3D, Dentsply Sirona, Charlotte, USA) (35, 36). Detailed information about the measurement methods of vasculopathy-related and SSc-specific clinical changes have been previously published (9, 12).

### Statistical analysis

Categorical variables were represented by frequency and percentages and analyzed by Chi-square test. Continuous variables were given as means and standard deviation or median and interquartile range. The normal distribution was examined with Shapiro–Wilk test and Student’s *t*-test was used for normally distributed data while Mann–Whitney-*U* test was used for non-normally distributed data. Pearson’s correlation coefficient was utilized for normally distributed variables and Spearman’s correlation coefficient was used for non-normally distributed variables. Statistical significance was assumed for *p* values<0.05 and statistical analyses were executed with SPSS version 27.0.

### Ethical approval

This study was approved by the local ethics committee (protocol number EK 29–361 ex 16/17) and was conducted in accordance with the Helsinki Declaration of 1975, as revised in 2013. All patients gave their written informed consent after being accurately informed about that clinical study.

## Results

Patients’ characteristics have been described elsewhere (9, 12). Median uric acid levels were 4.6 mg/dL (3.9–5.4 mg/dl 25th–75th percentile) in patients with lcSSc and 4.3 mg/dL (3.7–5.3 mg/dL 25th–75th percentile) in controls (*p* = 0.313). Mean disease duration was 7.1 ± 5.8 years in patients with lcSSc and 5.7 ± 3.2 years in controls (*p* = 0.191). No significant differences regarding amino acids, calciotropic or bone turnover parameters were observed between patients with lcSSc and controls. Only a trend toward higher levels of alanine, glutamic acid, glutamine, and PTH was observed in patients with lcSSc. Neither the rate of vitamin D insufficiency nor vitamin D deficiency differed between both groups (Table 1). At study inclusion, four lcSSc patients (10.5%) and two controls (5.3%) (*p* = 0.654) had a pre-existing osteoporosis, which was recorded by patient’s history, while 10 lcSSc patients (26.3%) and three controls (7.9%) (*p* = 0.065) had a pre-existing treatment against osteoporosis. Of those patients with a pre-existing therapy against osteoporosis, all had a peroral vitamin D supplementation, one lcSSc patient had additional denosumab and another lcSSc patient had additional alendronic acid. No patient had any pre-existing hyperparathyroidism.

**Table 1:** Bivariate analysis of amino acids and metabolic bone parameters.

	LcSSc (*n* = 38)	Controls (*n* = 38)	Value of *p*
Amino acids (μmol/l)			
Alanine, mean (±SD)	415.6 ± 93.3	379.4 ± 76.4	0.069
Asparagine, mean (±SD)	49.6 ± 9.5	49.2 ± 8.5	0.854
Aspartic acid, median (25–75th percentile)	15.1 (13.62–18.19)	15.6 (12.3–17.9)	0.852
Glutamine, mean (±SD)	619.6 ± 62.4	592.1 ± 73.2	0.082
Glutamic acid, median (25–75th percentile)	42.5 (33.6–55.3)	38.1 (30.7–44.1)	0.064
Glycine, median (25–75th percentile)	266.7 (233.4–302.7)	274.4 (220.8–331.3)	0.568
Histidine, mean (±SD)	73.1 ± 7.8	71.5 ± 8.7	0.405
Isoleucine, median (25–75th percentile)	28.4 (24.6–31.8)	27.9 (24.5–32.5)	0.852
Leucine, median (25–75th percentile)	102.2 (93.7–113.0)	104.0 (92.4–116.1)	0.755
Lysine, median (25–75th percentile)	171.6 (156.1–187.7)	176.0 (157.5–197.7)	0.461
Methionine, median (25–75th percentile)	25.8 (23.4–29.4)	26.3 (24.2–30.3)	0.648
Phenylalanine, median (25–75th percentile)	56.9 (54.5–65.4)	55.5 (52.2–61.9)	0.257
Serine, mean (±SD)	119.6 ± 16.1	124.4 ± 21.0	0.258
Taurine, median (25–75th percentile)	68.2 (56.7–93.1)	81.4 (61.9–110.9)	0.228
Threonine, median (25–75th percentile)	116.7 (104.4–134.9)	124.9 (102.5–137.3)	0.926
Tryptophan, median (25–75th percentile)	55.9 (51.4–66.0)	59.8 (52.0–67.5)	0.473
Tyrosine, median (25–75th percentile)	59.4 (51.7–70.6)	56.6 (49.8–64.7)	0.198
Valine, median (25–75th percentile)	199.7 (177.8–217.2)	197.0 (174.5–221.3)	0.917
Bone parameters			
PTH (pg/ml), median (25–75th percentile)	47.0 (36.1–56.7)	41.3 (31.3–52.0)	0.086
25-(OH)-D (ng/ml), mean (±SD) 25-(OH)-D < 20 ng/ml, *n* (%) 25-(OH)-D < 30 ng/ml, *n* (%)	26.97 ± 9.00 9 (23.68) 25 (65.79)	31.37 ± 14.79 7 (18.42) 19 (50.00)	0.122 0.574 0.163
Osteocalcin (ng/ml), mean (±SD)	25.68 ± 9.19	26.58 ± 11.30	0.704
P3NP (μg/ml), mean (±SD)	3.45 ± 0.84	3.29 ± 1.00	0.435

25-(OH)-D, 25-hydroxyvitamin D_3_; P3NP, procollagen-3 n-terminal peptide; PTH, parathyroid hormone.

### Correlations between amino acids and parameters of bone metabolism with endothelial dysfunction

**Figure 1:**
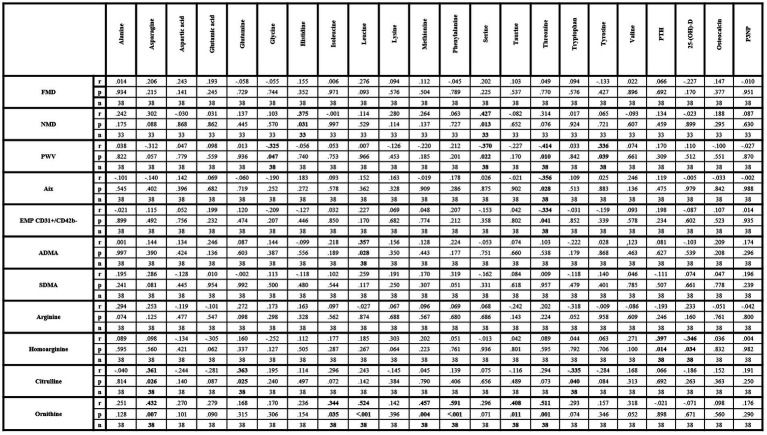
Correlation matrix of amino acids and metabolic bone parameters with parameters of endothelial dysfunction within lcSSc patients. 25-(OH)-D, 25-hydroxyvitamin D_3_; ADMA, asymmetric dimethylarginine; Aix, augmentation index; EMP, endothelial-derived microparticles; FMD, flow-mediated dilation; NMD, nitroglycerin-mediated dilation; P3NP, procollagen-3 n-terminal peptide; PTH, parathyroid hormone; PWV, pulse-wave velocity; SDMA, symmetric dimethylarginine. Bold values indicate statistical significance.

Significantly positive correlations were found between NMD, histidine and serine as well as between PWV and tyrosine. Significantly negative correlations between PWV, glycine and serine were also observed. Additionally, threonine revealed significantly negative correlations with PWV, augmentation index and EMP. Of the investigated arginine metabolites, significantly positive correlations were observed between ADMA and leucine, between citrulline, asparagine and glutamine, and between ornithine, asparagine, isoleucine, leucine, methionine, phenylalanine, taurine, and threonine. Significantly negative correlation between citrulline and tryptophan was also found. Neither SDMA nor arginine nor homoarginine correlated with any of the investigated amino acids. Homoarginine correlated significantly positively with PTH and negatively with 25-(OH)-D (Figure 1). Pathologic values of FMD, NMD and PWV were not associated with vitamin D insufficiency or deficiency (Table 2).

**Table 2:** Associations of parameters of endothelial dysfunction and SSc-specific clinical changes with vitamin D deficiency and insufficiency.

	25-(OH)-D < 20 ng/ml	25-(OH)-D < 30 ng/ml
FMD < 7%	0.736	0.593
NMD < 15.6%	0.592	0.171
PWV > 10 m/s	0.357	0.472
Present sclerodactylyl	0.871	0.743
Present telangiectasia	0.740	0.351
Present puffy finger	**0.046**	0.544
Early pattern	**0.040**	0.472
Active pattern	0.906	0.459
Late pattern	0.611	0.858
Mild periodontitis	0.176	0.326
Severe periodontitis	0.292	0.536
EUSTAR index ≥2.5	0.368	0.629

Asymptotic *p*-values of chi-square test. 25-(OH)-D, 25-hydroxyvitamin D_3_; EUSTAR, European Scleroderma Trials and Research Group; FMD, flow-mediated dilation; NMD, nitroglycerin-mediated dilation; PWV, pulse-wave velocity.

### Correlations between amino acids and parameters of bone metabolism with vasculopathy-related and SSc-specific changes

CSURI revealed a significantly positive correlation with isoleucine and leucine while MES did not show any correlation with amino acids. MRSS was significantly positive correlated with aspartic acid and glutamic acid and negatively with asparagine and methionine. Additional significantly negative correlations were observed between eGFR, alanine and tyrosine while protein/creatinine ratio did not correlate with any amino acid. The DETECT score correlated significantly positively with isoleucine, leucine, phenylalanine and valine, but neither the total score of the UCLA SCTC GIT 2.0 questionnaire nor the EUSTAR index revealed a correlation with any amino acid. Significantly negative correlations were found between threonine, plaque index and BOP as well as between BOP and asparagine.

**Figure 2:**
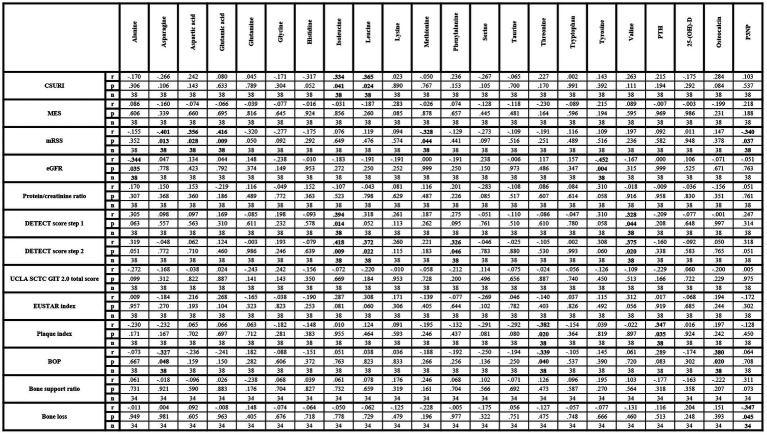
Correlation matrix of amino acids and metabolic bone parameters with clinical parameters within lcSSc patients. 25-(OH)-D, 25-hydroxyvitamin D_3_; BOP, bleeding on probe; CSURI, capillaroscopic skin ulcer risk index; eGFR, estimated glomerular filtration rate; EUSTAR, European Scleroderma Trials and Research Group; MES, microangiopathy evolution score; mRSS, modified Rodnan Skin Score; P3NP, procollagen-3 n-terminal peptide; PTH, parathyroid hormone. Bold values indicate statistical significance.

Of the investigated parameters of bone metabolism, significantly positive correlations were observed between PTH and plaque index as well as between BOP and osteocalcin. P3NP revealed significantly negative correlations with mRSS and alveolar bone loss. Vitamin D revealed no correlation with any vasculopathy-related or SSc-specific parameter (Figure 2). Vitamin D insufficiency was not associated with any of the investigated SSc-specific clinical changes, while significant associations were found between vitamin D deficiency, puffy finger and early pattern (Table 2).

## Discussion

Endothelial dysfunction plays an important role in the development of SSc-related vasculopathy while the promoting factors of endothelial dysfunction in SSc subtypes and its potential interaction with other pathophysiologic pathways still need to be elucidated. This study was able to demonstrate potential interactions between selected proteinogenic amino acids, calciotropic and bone turnover parameters as well as endothelial dysfunction, vasculopathy-related and clinical changes in lcSSc. However, none of the measured amino acids, calciotropic and bone turnover parameter revealed a significant difference between patients with lcSSc and controls. One possible explanation for the missing relevant difference of the measured parameters may be the early disease stage in the group of lcSSc patients, as none of the investigated subjects had typical SSc-related complications, like PAH, digital ulcers or renal crisis. Only two lcSSc patients (5.3%) had incipient interstitial lung disease. Compared to the study by Smolenska et al. (14), which also investigated amino acids, but in a patient cohort with lcSSc and diffuse cutaneous SSc, the rate of organ involvement was higher than in our study and the serum levels of many amino acids differed substantially compared to our results, suggesting a potential influence of the SSc subtype and activity. Previous studies on parameters of bone metabolism, especially on 25-(OH)-D, included patients with other SSc subtypes and with SSc-related complications, which may also influence the results on those parameters (37–39). In addition, it has to be noted that patients with primary Raynaud’s phenomenon may be not ideal controls for study measurements of parameters of bone metabolism, as even pediatric patients with primary Raynaud’s phenomenon may already have low serum vitamin D levels which could bias our results on parameters of bone metabolism (40).

Certain amino acids revealed significant correlations with parameters of endothelial dysfunction and some of the measured amino acids, including glutamic acid, glutamine, glycine, histidine, leucine, methionine, serine, or tyrosine, have been reported to have potential protective cardiovascular properties (41, 42). Interestingly, leucine may be also an inhibitor of nitric oxide synthase (43). These data are, however, controversial to our findings. On the one hand, positive correlations were found between histidine and NMD and between leucine and ADMA, while negative correlations were observed between glycine, serine and PWV. These results are indicating potential vasoprotective effects for glycine, serine and histidine and potential damaging effects for leucine, which are in accordance with previous studies (41–43). On the other hand, glutamic acid revealed no correlations with any of the measured parameters of endothelial dysfunction. Glutamine and methionine were only positively correlated with citrulline and ornithine, while tyrosine was positively correlated with PWV, although inverse associations have been reported (41). Interestingly, threonine revealed also negative correlations with parameters of arterial stiffness and with EMP suggesting a potential unknown vasoprotective effects. However, the underlying interactions need to be investigated by further studies. Besides ADMA, only citrulline and ornithine correlated with other amino acids, but not arginine, homoarginine or SDMA. The correlations between asparagine, glutamine, citrulline, and ornithine may be expectable due to interactive metabolic processes between these amino acids (44). Conversely, especially the correlations of ornithine with isoleucine, leucine, methionine, phenylalanine, taurine and threonine are of interest as these amino acids have no known metabolic pathway interactions. Potential changes in other metabolic pathways, like polyamine metabolism, which were not investigated in this study, may contribute to these correlations (45, 46). Finally, significant correlations between homoarginine, PTH and 25-(OH)-D only were observed. This might indicate a potential interaction, possibly via osteo−/chondrogenic transformation of vascular smooth muscle cells and subsequent vascular calcification, as previously reported (47). However, this effect may be marginal or undetectable via the systemic detection or the current study design as neither PWV nor augmentation index correlated with PTH or 25-(OH)-D.

CSURI correlated with isoleucine and leucine suggesting a contribution of capillary damage by these chemically similar amino acids, potentially via an interaction of ADMA. At least leucine may act as an inhibitor of nitric oxide synthase and ADMA was shown to correlate with CSURI in a previous study (12, 43). Overall, the association of amino acids with capillary changes seems to be low, as no other amino acid correlated neither with CSURI nor with MES. Skin sclerosis, which was evaluated by mRSS, revealed correlations with asparagine, aspartic acid, glutamic acid and methionine. Elevated glutamate, an anion of glutamic acid, and decreased levels of asparagine have been associated with calcinosis and extensive scleroderma (14). Our data are comparable to these results, as asparagine and glutamine revealed negative correlations while aspartic acid and glutamic acid showed positive correlations with mRSS. Since both, asparagine and glutamine, are reported to be mediators of immune function, dysbalance of their metabolic turn-over may impair immunoregulation with subsequent contribution to skin sclerosis (48, 49). In addition, lower methionine levels may promote skin sclerosis as methionine may affect inflammatory and fibrotic processes (50).

Negative correlations between eGFR, alanine and tyrosine but not with urinary total protein/creatinine ratio were also observed. Alanine was previously described as a potential biomarker in patients with acute kidney injury and showed associations with the prevalence of chronic kidney disease (51, 52). Moreover, impaired kidney function leads to reduced conversion of phenylalanine to tyrosine (53). Thus, these associations seem to be a result of impaired kidney function rather than specific SSc-related changes in the kidney. Nevertheless, whether these alterations may reflect kidney dysfunction associated with vasculopathy in SSc patients need to be elucidated in more detail.

Subclinical PAH, as evaluated by the DETECT score, correlated with isoleucine, leucine, phenylalanine and valine. Again, this might, at least partially, be due to potential interactions via ADMA, since ADMA previously showed associations with the DETECT score (12). On the other hand, associations of higher phenylalanine and valine levels with pulmonary involvement were observed in previous studies, suggesting therefore a potential interaction between selected amino acids with PAH (14, 54). Interestingly, no amino acid was associated with gastrointestinal involvement evaluated by UCLA SCTC GIT 2.0 questionnaire or with disease activity as measured by EUSTAR index. While potential involvement of amino acids in gastrointestinal changes and disease activity cannot be excluded, as only two of many potential parameters were evaluated, further studies are needed to evaluate other parameters of gastrointestinal involvement and disease activity.

Further potential associations between threonine, plaque index and BOP were observed, suggesting anti-inflammatory effects of threonine. Negative correlations of P3NP with mRSS and bone loss indicating that P3NP, as a marker of collagen synthesis in bone and muscles, may have an impact on collagen turn-over in skin and alveolar bone (55). Additionally, positive associations between PTH and plaque index were observed which may be explained by higher serum and salivary phosphorus levels, which were found in patients with hemodialysis and elevated PTH levels (56). The fact that vitamin D did not show any correlation with vasculopathy-related or clinical changes while vitamin D deficiency was associated only with puffy finger and capillaroscopic early pattern, might indicate only marginally influence of 25-(OH)-D in early stage vasculopathy. Similarly, associations between osteocalcin and BOP seem to be by chance without relevant influence on periodontal disease, while no correlation between osteocalcin and bone loss or bone support ratio was observed.

One limitation of this study is the exploratory design with measurements of many parameters without adjustment for multiple testing which allows therefore only some hypothesis generation. Additional limitations are the relatively small sample size and the recruitment of patients with Raynaud’s phenomenon as controls that might affect the results on metabolic parameters (40). However, it has to be noted that this study is a sub-study of a previous investigation on endothelial dysfunction in patients with lcSSc in which the inclusion of patients with Raynaud’s phenomenon as controls was adequate. Strengths are homogenous cohorts with age-, race-and sex-matched controls and absent end-stage vasculopathic changes.

In conclusion, certain amino acids may be associated with endothelial function and may contribute to vasculopathy-related and clinical changes in lcSSc patients with early-stage vasculopathy. However, their clinical relevance in SSc, especially as predictors of SSc-related complications, and potential interaction with other metabolic parameters needs to be further elucidated. The relevance of the investigated calciotropic and bone turnover parameters on endothelial dysfunction and on vasculopathy-related and clinical changes seems to be marginally. Nevertheless, further studies will probably reveal potential interactions of metabolic parameters in SSc.

## Data availability statement

The original contributions presented in the study are included in the article/supplementary material, further inquiries can be directed to the corresponding author.

## Ethics statement

The studies involving human participants were reviewed and approved by Ethics committee of the Medical University of Graz. The patients/participants provided their written informed consent to participate in this study.

## Author contributions

PJ and FH contributed to study design, study measurements, and funding acquisition. PJ contributed to writing of the manuscript and data analysis. AM, HS, BA, GW, BO-P, VF, and GK contributed to study measurements, data interpretation, and reviewing of the manuscript. BO and FM-F contributed to data interpretation and reviewing of the manuscript. MB contributed to reviewing of the manuscript and supervised the project. All authors contributed to the article and approved the submitted version.

## Funding

This study was funded by Actelion Pharmaceuticals, Vienna, Austria. The funder was not involved in the study design, collection, analysis, interpretation of data, the writing of this article, or the decision to submit it for publication.

## Conflict of interest

The authors declare that the research was conducted in the absence of any commercial or financial relationships that could be construed as a potential conflict of interest.

## Publisher’s note

All claims expressed in this article are solely those of the authors and do not necessarily represent those of their affiliated organizations, or those of the publisher, the editors and the reviewers. Any product that may be evaluated in this article, or claim that may be made by its manufacturer, is not guaranteed or endorsed by the publisher.
